# Traditional knowledge of plants used in hunting and fishing practices among Baka hunter-gatherers of eastern Cameroon

**DOI:** 10.1186/s13002-022-00571-3

**Published:** 2023-01-03

**Authors:** Evariste Fedoung Fongnzossie, Marlène Tounkam Ngansop, Takanori Oishi, Achille Bernard Biwole, Elvire Hortense Biye, Mitsuo Ichikawa

**Affiliations:** 1 Advanced Teacher’s Training College for Technical Education, Laboratory of Forest and Wood Resources Valorisation, University of Douala, P.O. Box, 1872 Douala, Cameroon; 2 Advanced Teacher’s Training College for Technical Education Laboratory of Process Engineering, University of Douala, P.O. Box, 1872 Douala, Cameroon; 3 African Studies Centre, Tokyo University of Foreign Studies, 3-11-1 Asahicho,, Fuchu, Tokyo 183-8534 Japan; 4 Department of Plant Biology, Faculty of Science, University of Yaoundé I, P.O. Box: 812, Yaoundé, Cameroon; 5 Centre for African Area Studies, Kyoto University, Kyoto, 606-8501 Japan

**Keywords:** Baka hunter-gatherers, Hunting, Fishing, Ethnobotanical knowledge, Cameroon

## Abstract

**Background:**

Baka hunter-gatherers have a well-developed traditional knowledge of using plants for a variety of purposes including hunting and fishing. However, comprehensive documentation on the use of plants for hunting and fishing in eastern Cameroon is still lacking.

**Method:**

This study aimed at recording plants used for hunting and fishing practices, using focus group discussion, interviews and field surveys with 165 Baka members (90 men and 75 women) of different age groups in 6 villages.

**Results:**

The most frequent techniques used for hunting and fishing are the use of animal traps, fishing lines, dam fishing, hunting with dogs and spear hunting. We recorded a total of 176 plant species used in various hunting practices, the most frequently cited one being *Zanthoxylum gilletii* (De Wild.) P.G.Waterman*, Greenwayodendron suaveolens* (Engl. & Diels) Verdc.*, Microcos coriacea* (Mast.) Burret*, Calamus deërratus* G.Mann & H.Wendl*.* and *Drypetes* sp. These plants are used for a variety of purposes, most frequently as hunting luck, psychoactive for improving the dog’s scent and capacity for hunting, materials for traps, and remedies for attracting animals and for making the hunter courageous.

**Conclusion:**

Plants used for hunting purposes here are embedded in a complex ecological and cultural context based on morphological characteristics, plant properties and local beliefs. This study provides a preliminary report and leaves room for further investigations to improve the documentation of the traditional knowledge systems of the studied community.

## Introduction

Baka hunter-gatherers heavily depend on wild forest resources (plants, animals) to meet their subsistence and cash income needs. Some studies in several sites have shown that they have a well-developed traditional knowledge of using plants for a variety of purposes including not only for direct material uses as food, medicines, craft and building materials hunting, and fishing but also for religious practices [1–8]. Hunting and fishing by the Baka hunter-gatherers are very important activities from ecological, social and cultural points of view, and bushmeat is among their most preferred food [9]. Although they are traditionally spear hunters [10], they have experienced through time a diversity of techniques and methods for hunting and fishing including snare hunting for medium-sized mammals, mouse traps, use of fire to smoke prey animals out of burrows, dam fishing, fish-poisoning, hunting with dogs, machetes ad catapults, as well as hunting with shotguns, from sedentary or migratory camps, etc. [11–16].

Various studies have also reported the use of plants and plant products for fishing and hunting. Previous studies reported up to 325 fish poison species used in tropical Africa, the most frequently used being *Tephrosia vogelii* Hook.f.*, Mundulea sericea* (Willd.) A.Chev.*, Euphorbia tirucalli* L.*, Gnidia kraussiana* Meisn.*, Adenia lobata* (Jacq.) Engl and *Balanites aegyptiaca* (L.) Delile [17]. Many of these species were also reported as used for preparing arrow poisons and traditional medicine. Hunting of the Baka people not only involves the direct use of poisonous plants and weapons such as gun, nets, spear, bows and traps. It also involves the use of dogs, especially the use of plant medicine for improving the dogs’ hunting ability, and for a variety of hunting rituals. While ethnobotanical knowledge of the Baka hunter-gatherers has been investigated by several authors, comprehensive documentation on the use of plants for hunting is still lacking.

In the face of the recent shift in the Bakas’ subsistence activity from hunting and gathering to farming, the change in their lifestyle and the development of education support projects, anthropologists and activists working for indigenous issues have expressed their concern about the risks of degradation of traditional ecological knowledge among Baka hunter-gatherers. Indeed, it seems that fewer Baka people are now involved in traditional hunting and gathering activities. [18] emphasize that the creation of national park and forest concessions are the factors that limited the access of Baka members to hunting. However, previous studies show that a number of socio-economic variables influence traditional ecological knowledge among indigenous communities. These variables include age, gender, consumerism, occupation, and psychosocial variables [19–24].

.These studies, depending on the scale of analysis, pointed out significant differences on national and continental levels. On the global level, however, no significant difference was reported between women and men. Concerning the age variable, several studies reveal that youth are reported to show a greater diversity of plant knowledge [22, 25]. The present research aims at documenting the diverse uses of plants in hunting practices among the Baka community members in Eastern Cameroon. We hypothesize that the ethnobotanical hunting knowledge of Baka hunter-gatherers is rich and varies with age and gender.

## Material and methods

### Study site

**Fig. 1:**
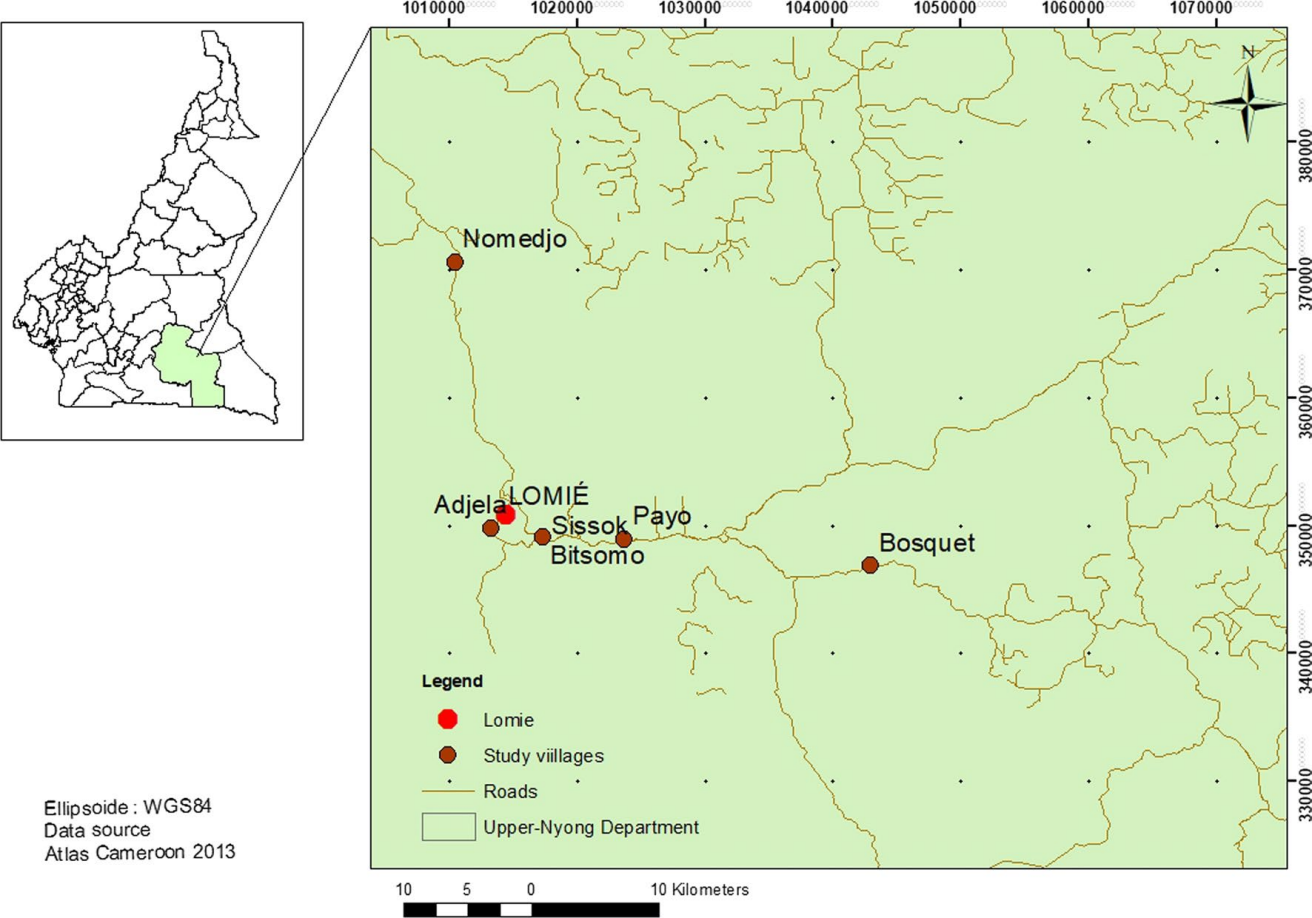
Location of study villages

The study was conducted in six villages along the road from Abong Mbang to Messok: Bitsomo (3.14785 N, 13.65072E), Nomedjo (3.34169 N, 13.59048 E), Adjela Baka (3.15397 N, 13.61487 E), Sissok (3.147857 N, 13.65072E), Payo (3.14569 N, 13.70801E) and Bosquet (3.12655 N,13.88085E) (Fig. 1). These villages are located in the Lomie council, in the Haut Nyong Division of, East Cameroon Region. The total population of the council is approximately 19,000 inhabitants [26]. The local population consists of four main ethnic groups: Zime, Kako, Ndjeme and the Baka, living along the side road and speaking the Baka language. They are mainly Christians and Muslims [26]. The predominant forms of their livelihoods are shifting cultivation, cacao garden cultivation, hunting, fishing and gathering. Various, NTFPs (Non-Timber Forest Products) are collected and sold.

The vegetation of the area is part of the camerouno-congolian forest consisting of a semi-deciduous forest comprised of a majority of *Malvaceae* and *Ulmaceae* [27, 28].

The area is subject to a Guinean equatorial climate with four seasons divided as follows: a long dry season from December to mid-March; a short rainy season from mid-March to June; a short dry season in July–August; a long rainy season from August to November. The mean annual rainfall varies from 1500 to 2000 mm, with an average temperature of 24 °C.

## Research methods

### Sampling and data collection approach

The ethnobotanical approach applied in this study used focal group discussion, interviews and field surveys in the forest, with 165 Baka members from the six study villages (Table 1). These villages were chosen based on the presence of an important Baka community and the prior consent given by the Chief of the village. Respondents in each village were chosen at random, based on their willingness to participate in the research.

**Table 1:** Sociodemographic characteristics of respondents

Characteristics	Number of respondents	Percentage
Gender		
Men	90	54.55
Women	75	45.45
Age group		
10–20 years	36	21.82
20–30 years	55	33.33
30–40 years	27	16.36
40–50 years	19	10.91
50–60 years	14	8.48
+ 60 years	15	9.09
Number of years of school attendance		
No school attendance	17	10.30
1–3 years	62	37.58
4–6 years	71	43.03
7–10 years	15	9.91

During the focal group discussion, it was clearly explained to the community members that the objective of the study was to record the plants used for hunting and fishing practices according to age and gender to obtain their prior informed oral consent. Individual interviews were conducted to gather information on their experience of using plants in hunting and fishing practices. Respondents were asked about their age, gender, hunting methods employed, local names of plants used in hunting activities, parts used, and usage. To obtain effective participation of respondents, interviews were conducted in the local language with the help of local translators.

The interviews were followed by fieldwork in the forest, which gave an opportunity for more discussions with respondents. This was also an opportunity to observe and gather information on the plant species free-listed by respondents. Plants named during the interviews were identified in situ in the field using available floral reference literature [29–32]. Plants that were spontaneously found when walking in the forest were also considered and information on their uses was recorded. For each unidentified plant species cited during the interview in the village, a specimen was collected, pressed and dried, and their identification was confirmed at the Cameroon National Herbarium in Yaoundé (YA). The voucher specimen was kept at Millennium Ecological Museum Herbarium and at the National Herbarium in Yaounde. Some of the plants listed by respondents during the interviews were not found during the forest walks and remained unidentified. These plants referred to as ethnospecies in the present study also included those that were known in their uses category, but the respondent was not able to remember the vernacular name.

### Data analysis

Simple descriptive statistics were applied to represent and list the number and percentage of species of plants and plant parts used. The floral list of plants cited by respondents was grouped based on their age and gender. The frequency of citation (F) for each hunting and fishing technique and species was calculated. It corresponds to the ratio between the number of respondents (n) having cited the technique or species and the total number of respondents (N):

$$F = n/N \times 100.$$

The magnitude of plant knowledge among each group (represented by the total number of plants cited) was used as a measurement of ethnobotanical knowledge. NNESS similarity index between the lists of plant species cited by each group was used as means of comparison.

This index is used to compare with minimum bias the degree of similarity of two samples (*i* and *j*) on the basis of an identical data size *k* randomly selected from each sample. The similarity between the two samples (*i* and *j*) is expressed by the Morisita-Horn index and by its generalization, the NNESS index, which is a variant of the NESS index [33]. The formula is given below and was computed using the software BiodivR 1.0 [34].

$${\text{NNESS}}\;ij/k = \frac{{{\text{ESS}}\;ij/k}}{{\left( {{\text{ESS}}\;ii/k + {\text{ ESS}}\;jj/k} \right)/{2}}}$$

where ESS *ij/k* is the expected number of species shared for random draws of* k* specimens from sample *i* and *k* specimens from sample *j*. The more the value is close to 1, the more the pairs of species lists compared are floristically similar. Values above 0.5 can denote a great number of similar species shared by the two lists.

## Results

### Hunting techniques

**Fig. 2:**
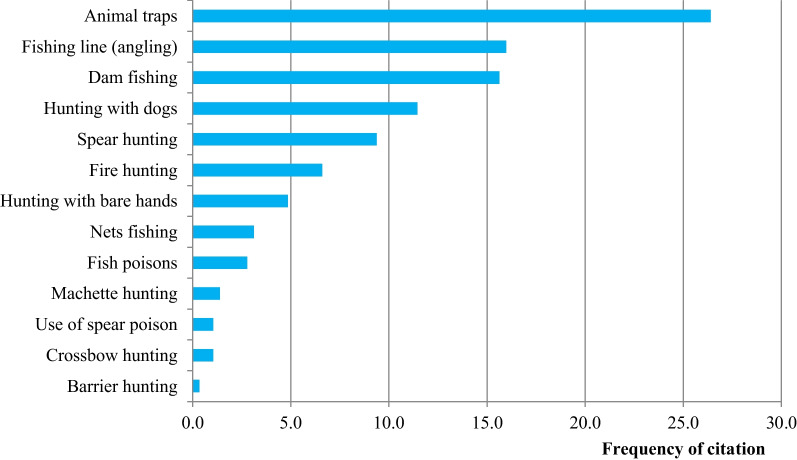
Frequently used hunting techniques

The results showed a total of 13 hunting techniques used by the Baka hunter-gatherers. The most frequently used techniques are animal traps, fishing lines, dam fishing, hunting with dogs and spear hunting (Fig. 2).

### Plant species used in hunting practices

**Fig. 3:**
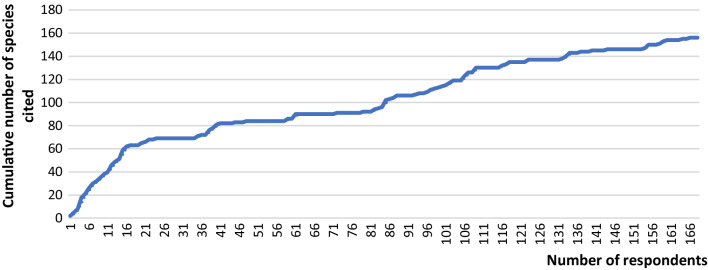
Cumulative diagram of plant species listed by interviewees

A total of 176 plant species were recorded as used in the various hunting practices from the interviews with the Baka. The cumulative diagram of plant species recorded showed that the sampling size was adequate, as new species were hardly added despite the increase in the number of persons interviewed (Fig. 3).

The most frequently cited species were *Zanthoxylum gilletii* (De Wild.) P.G.Waterman*, Greenwayodendron suaveolens (Engl. & Diels) Verdc., Microcos coriacea* (Mast.) Burret*, Calamus deerratus* G.Mann & H.Wendl and *Drypetes* sp. (Table 2). While the rattan species identified during the survey was *Calamus deerratus*, respondents insisted that all rattan species can be used as well.

**Table 2:** List of recorded plant species used by Baka hunter-gatherers in hunting practices

Scientific name	Familly	Vernacular name (in Baka)	Voucher number	Part used	Usage	Frequency of citation
*Zanthoxylum gilletii *(De Wild.) P.G.Waterman	Rutaceae	Bolongo, Ntawolo, Apouo'o, Njo'o	Nana P. 36	Stem, leaves	Stems are used as fish poison and leaves are used to bring luck	7.85
*Greenwayodendron suaveolens (Engl. & Diels) Verdc*	Annonaceae	Botounga	de Wilde J.J.F.E. 7940	Stem, bark	Barks are used to bring luck and to attract animals, stems are used as trap lever, to attract animals and to bring luck	7.74
*Microcos coriacea *(Mast.) Burret	Malvaceae	Bokou	Webb J. 05	Fruit, bark	Fruits and barks are used as fish poison	6.14
*Rattan*	Arecaceae	kpongo	Raynal J. 10,548	Stem	Stems are used to make baskets to carry catches and to make lobster pot	4.89
*Drypetes sp.*	Putranjivaceae	Kpwasso'o	Letouzey R. 12,210	Leaves, stem	Leaves are used to increase dog’s hunting performance or to bring luck, stems are used as trap lever	3.75
*Microdesmis puberula Hook.f. ex Planch*	Pandaceae	Pipi/Fifi	Lejoly J. 899	Stem	Stems are used as trap lever	3.64
*Desbordesia glaucescens *(Engl.) Tiegh	Irvingiaceae	Solia	Nkongmeneck B.-A. 392	Bark, stem	Barks used to bring luck, attract animals or get courage, stems used as trap lever	2.84
*Ataenidia conferta *(Benth.) Milne-Redh	Marantaceae	Boboko	Breteler F.J. 789	Leaves	Use in chasing water during dam fishing	2.73
*Diospyros hoyleana *F.White	Ebenaceae	Bokembé	Letouzey R.573	Stem	Stems are used as trap lever, Barks use to have courage and to become invisible, stem used to make spear	2.62
*Massularia acuminata (G.Don) Bullock ex Hoyle*	Rubiaceae	Mindo	Thomas D.W. 6939	Stem, leaves	Stems used as trap lever; leaves used to increase dog’s hunting performance	2.05
*Asystasia gangetica *(L.) T.Anderson	Acanthaceae	Apouo'o	Thomas D.W. 6769	Leaves	Leaves are used as fish poison	1.82
*Haumania danckelmaniana *(J.Braun & K.Schum.) Milne-Redh	Marantaceae	Kpwasele	Letouzey R. 3652	Leaves, stem	Leaves and stems are used to bring luck, stems are also used as trap lever and to attract animals, leaves are used in chasing water during dam fishing and to give drug to dog's	1.71
*Heisteria zimmereri *Engl	Olacaceae	Molomba, Modobalomba		Stem, bark	Stems are use as trap lever; bark are used to bring luck	1.59
*Landolphia heudelotii *A.DC	Apocynaceae	Mokendjo	Breteler F.J. 2158	Leaves, stem, fruit	Leaves are used to get courage, Stems are used to make spear, fruits are used to increase dog’s hunting performance	1.59
*Sloetiopsis usambarensis Engl*	Moraceae	Ndoundoun	Cheek M. 8846	Stem, root, bark and leaves	Stems and roots are used as trap lever, barks are used to bring luck, leave are used to get courage	1.48
*Anonidium mannii *(Oliv.) Engl. & Diels	Annonaceae	Gbwé	Thomas D.W. 7641	Bark, leaves	Barks are used to be invisible; leave are used to attract animals	1.37
*Brenania brieyi *(De Wild.) E.M.A.Petit	Rubiaceae	Molondjo	Thomas D.W. 8743	Bark	Barks are used to increase dog’s hunting performance	1.37
*Erythrophleum suaveolens *(Guill. & Perr.) Brenan	Fabaceae	Mbanda	Sonké B. 999	Stem, bark	Stems and barks are used to bring luck, to increase dog's hunting performance, to avoid to meet ferocious animals and as fish poison	1.37
*Tabernaemontana crassa Benth*	Apocynaceae	Pandor	Thomas D.W. 7575	Stem, bark, leaves	Stems are used to attract animals, Barks and leaves are used to bring luck	1.37
*Alchornea floribunda *Müll.Arg	Euphorbiaceae	Yando	Nemba 104	Root, leaves, stem	Root and stem are used to bring luck, leaves are used to increase dog’s hunting performance	1.02
*Baillonella toxisperma *Pierre	Sapotaceae	Mabé	Letouzey R. 2805	Stem, fruit pulp, bark	Stems are used to make gun and to become invisible, fruit pulps are used as fish poison and barks are used to attract animals	1.02
*Strophanthus gratus (Wall. & Hook.) Baill*	Apocynaceae	Nea	Onana J.-M. 115	Bark, sap	Barks and saps are used as spear poison	0.91
*Terminalia superba Engl. & Diels*	Combretaceae	Ngoulou	Thomas D.W. 6065	Stem	Stems are used as trap lever	0.91
*Xylopia sp.*	Annonaceae	Koulou, Mpoulou	Thomas D.W. 4878	Stem	Stems are used to become invisible, to bring chance and protection and as fish poison	0.91
*Acacia pennata *(L.) Willd	Fabaceae	Mpala	Letouzey R. 10,603	Leaves	Leaves are used to increase dog’s hunting performance	0.8
*Annickia chlorantha (Oliv.) Setten & Maas*	Annonaceae	Efoué	Endengle E. 87	Stem	Stems are used as trap lever	0.8
*Cylicodiscus gabunensis *Harms	Fabaceae	bolouma	Letouzey R. 10,063	Bark	Barks are used to increase dog’s hunting performance	0.8
*Barteria nigritiana *Hook.f	Passifloraceae	Fambo	Letouzey R. 12,458	Bark	Barks are used to have luck and to attract animals	0.68
*Manniophyton fulvum Müll.Arg*	Euphorbiaceae	Coussa		Leaves, stem	Leaves are used to have luck; stems are used to make baskets to carry catches	0.68
*Rourea obliquifoliolata Gilg*	Connaraceae	Mongassa	Koufani A. 32	Stem, bark	Stems are used to make spear and as trap lever, barks are used to get courage	0.68
*Scleria boivinii Steud*	Cyperaceae	Tiyéyé	Kaji M. 254	Stem, leaves, root	Stems, leaves and roots are used to bring luck	0.68
*Alstonia boonei *De Wild	Apocynaceae	Gouga	Nana P. 398	Sap,bark, stem	Saps are used to bring luck, barks are used to attract animals, young stems are used to make shaft	0.57
*Cleistopholis glauca *Pierre ex Engl. & Diels	Annonaceae	Molombo	Tamaki 79	Bark	Barks used as fish poison	0.57
*Geophila cordifolia *Miq	Rubiaceae	Djakelem	Letouzey R. 13,922	Leaves	Leaves are used to bring luck	0.57
*Klainedoxa gabonensis *Pierre ex Engl	Irvingiaceae	Mongasa	Thomas D.W. 6761	Stem, bark	Stems are used to make spear; barks are used to have power	0.57
*Leptactina congolana *(Robbr.) De Block	Rubiaceae	Nyambanou	Manning S.D. 1044	Leaves	Leaves are used to become invisible	0.57
*Triplochiton scleroxylon K.Schum*	Malvaceae	Gwado	Mpom B. 245	Bark, leaves	Barks are used as spear poison and to bring luck, leaves are used to bring luck	0.57
Turraeanthus africanus (Welw. ex C.DC.) Pellegr	Meliaceae	Assama	Cheek M. 9048	Bark, leaves	Barks and leaves are used as fish poison	0.57
*Albizia sp.*		Esa'a	Villiers J.-F. 4750	Bark	Barks are used to attract animals	0.46
*Capsicum frutescens *L	Solanaceae	Alamba	Westphal 10,028	Root, fruit, stem	Roots are used to bring luck, stems are used to attract animals, fruits are used to take the animal out of the hole	0.46
*Clausena anisata *(Willd.) Hook.f. ex Benth	Rutaceae	Toukoussa	Letouzey R. 4350	Stem	Stems are used to bring luck/leaves are used to avoid to meet ferocious animals	0.46
*Cleistopholis patens *(Benth.) Engl. & Diels	Annonaceae	Kiyo afane	Letouzey R. 10,523	Bark	Barks are used to get courage	0.46
*Irvingia gabonensis *(Aubry-Lecomte ex O'Rorke) Baill	Irvingiaceae	Péké	Letouzey R. 11,401	Bark, stem	Barks are used to attract animal; stems are used to make fishing rod	0.46
*Myrianthus arboreus P.Beauv*	Urticaceae	Ngata	Nana P. 368	Leaves	Leaves are used to attract animals	0.46
*Pentaclethra macrophylla Benth*	Fabaceae	Balaka	Leeuwenberg A.J.M. 9870	Stem, bark, fruit	Stems are used as trap lever; barks are use as fish poison and fruits are used to attract animals	0.46
*Piptadeniastrum africanum (Hook.f.) Brenan*	Fabaceae	Koungou	Nemba 122	Bark	Barks are used to bring luck and to increase dog's hunting performance	0.46
*Pterocarpus soyauxii Taub*	Fabaceae	Nguèlè	Bos 3272	Stem, bark	Barks are used to bring luck; stems are used as trap lever	0.46
*Ricinodendron heudelotii (Baill.) Heckel*	Euphorbiaceae	Gobo	Leeuwenberg A.J.M. 5970	Bark	Barks are used to attract animals and to bring luck in fishing	0.46
*Strombosia pustulata Oliv*	Olacaceae	Bobongo	de Wilde J.J.F.E. 8218	Stem	Stems are used as trap lever	0.46
*Tetrapleura tetraptera (Schumach. & Thonn.) Taub*	Fabaceae	Djaga	Mpom B.15	Bark	Barks are used to increase dog’s hunting performance	0.46
*Anopyxis klaineana *(Pierre) Engl	Rhizophoraceae	Eboma		Bark	Barks are used to attract animals and to have luck	0.45
*Afrostyrax lepidophyllus *Mildbr	Huaceae	Nguimba	Letouzey R. 18,387	Bark	Barks are used like hunt poison	0.34
*Bombax buonopozense *P.Beauv	Malvaceae	Ntombi	Villiers J.-F. 703		Used to bring luck	0.34
*Diospyros sp.*	Ebenaceae	Bokembé	Thomas D.W. 7209	Stem	Stems used as trap lever, Barks use to have courage and to become invisible, stem used to make spear	0.34
*Entandrophragma cylindricum *(Sprague) Sprague	Meliaceae	Boyo	Breteler F.J. 2697	Stem	Stems are used to make gun	0.34
*Lepidobotrys staudtii *Engl	Lepidobotryaceae	Wassassa	Letouzey R. 3900	Bark	Barks are used to increase dog’s hunting performance	0.34
*Leptaspis zeylanica Nees ex Steud.//Leptaspis coclheata*	Poaceae	Dingwélingwé	Jacques-Félix H. _&27	Leaves	Leaves are used to get courage,	0.34
*Panda oleosa Pierre*	Pandaceae	Kana	Letouzey R. 43,237	Bark, stem	Barks are used to attract animals; stems are used as trap lever	0.34
*Psychotria cyanopharynx K.Schum*	Rubiaceae	Mbongo	Letouzey R. 10,554	Leaves, fruit	Leaves are used to increase dog's hunting performance; fruits are used as fish poison	0.34
*Strombosiopsis tetrandra Engl*	Olacaceae	Bossiko	de Wilde J.J.F.E. 8250	Fruit, stem	Fruits are used to increase dog's hunting performance; stems are used as trap lever	0.34
*Ficus platyphylla *Delile	Moraceae	Ekom	Villiers J.-F. 1398	Stem	Stems are used as trap lever	0.33
*Bikinia letestui (Pellegr.) Wieringa*	Fabaceae	Ngassa	Cheek M. 8823	Stem	Stems are used as trap lever	0.23
*Campylospermum elongatum *(Oliv.) Tiegh	Ochnaceae	Kpwadjelé	Asonganyi J.N. 229	Leaves	Leaves are used to bring luck and to attract animals	0.23
*Celtis zenkeri *Engl	Cannabaceae	Ngombé	Amshoff G.J.H. 6227	Stem	Stems are used as trap lever	0.23
*Diospyros crassiflora *Hiern	Ebenaceae	Lémbé	Letouzey R. 4785	Stem	Stems are used as trap lever	0.23
*Entandrophragma utile *(Dawe & Sprague) Sprague	Meliaceae	Gbwokoulou	Letouzey R. 12,017	Stem	Stems are used to make gun	0.23
*Eremospatha wendlandiana *Dammer ex Becc	Arecaceae	Nkao'o	Letouzey R. 4151	Stem	Stems are used to make lobster pot	0.23
*Fire*		Wa'a			Fires are used to take the animal out of the hole	0.23
*Gilbertiodendron dewevrei *(De Wild.) J.Léonard	Fabaceae	Bokou	Letouzey R. 1736	Fruit, bark	Fruits and barks are used as fish poison	0.23
*Hylodendron gabunense *Taub	Fabaceae	Lando'o	de Wilde J.J.F.E. 8214	Stem	Stems are used as trap lever	0.23
*Irvingia sp.*	Irvingiaceae	Nto'o	Bullock S.H. 520	Bark	Barks are used to become invisible	0.23
*Manihot esculenta *Crantz	Euphorbiaceae	Boma	Westphal 8933	Tuber	Tubers are used to increase dog's hunting performance	0.23
*Mimosa invisa Mart. ex Colla*	Fabaceae	Nkenkeguili	Westphal 9836	Leaves	Leaves are used to bring luck	0.23
*Musanga cecropioides R.Br. ex Tedlie*	Urticaceae	Kombo	Leeuwenberg A.J.M. 6713	Bark	Barks are used to bring luck	0.23
*Pericopsis elata (Harms) Meeuwen*	Fabaceae	Mobaye	Letouzey R. 12,099	Bark	Barks are used to get courage	0.23
*Pollia condensata C.B.Clarke*	Commelinaceae	Salabimbi	Kengué 11	Leaves, stem	Leaves and stems are used to bring luck	0.23
*Pycnanthus angolensis (Welw.) Warb*	Myristicaceae	Etingue	Letouzey R. 178	Bark	Barks are used to attract animals	0.23
*Terminalia sp.*	Combretaceae	Ngindi	Nana P. 400	Stem	Stems are used as trap lever	0.23
*Triplophyllum protensum* (Sw.) Holttum	Tectariaceae	Ndélé, Edélé		Leaves	Leaves are used to bring luck	0.23
*Adenia cissampeloides *(Planch. ex Hook.) Harms	Passifloraceae	Poulou	Letouzey R. 9250	Stem	Stems are used to bring luck	0.11
*Aframomum sp.*	Zingiberaceae	Nji'i	Thomas D.W. 3054	Leaves	Leaves are used to increase dog’s hunting performance	0.11
*Anthocleista schweinfurthii *Gilg	Gentianaceae	Eba	Bos 5686	Bark	Barks are used to bring luck	0.11
*Capsicum sp.*	Solanaceae	Alamba	Westphal 9884	Root, fruit, stem	Roots are used to bring luck, stems are used to attract animals, fruits are used to take the animal out of the hole	0.11
*Ceiba pentandra *(L.) Gaertn	Malvaceae	Baoba	Bamps P.R.J. 1448	Bark	Barks are used to bring luck	0.11
*Celtis adolfi-friderici *Engl	Cannabaceae	Kakala	Breteler F.J. 2456	Bark	Barks are used to bring luck	0.11
*Cola nitida* (Vent.) Schott & Endl	Malvaceae	Bolouga	Nkongmeneck B.-A. 106	Fruit	Fruits are used to get courage	0.11
*Cordia africana *Lam	Boraginaceae	Mbabi	Satabié B. 173	Bark	Barks are used to become invisible	0.11
*Coula edulis *Baill	Olacaceae	Npkombo	de Wilde J.J.F.E. 8218	Stem, bark	Stems are used to stop water during dam fishing, barks are used to bring luck and to get courage	0.11
*Detarium macrocarpum *Harms	Fabaceae	Mili	Biholong M. 50	Fruit	Fruits are used as fish poison	0.11
*Distemonanthus benthamianus*		Sele	de Wilde J.J.F.E. 7969		Used to become invisible	0.11
*Drypetes gossweileri *S.Moore	Putranjivaceae	Ebomaka	Letouzey R. 4423	Leaves	Leaves are used to bring luck	0.11
*Elaeis guineensis *Jacq	Arecaceae	Bila		Fruit	Fruits are used to bring luck	0.11
*Entandrophra sp.*	Meliaceae	Eboyo	Nemba 481	Leaves	Leaves are used to increase dog's hunting performance	0.11
*Gambeya africana *(A.DC.) Pierre	Sapotaceae	Sasagoulou	Letouzey R. 9282	Leaves	Leaves are used to bring luck	0.11
*Hunteria umbellata *(K.Schum.) Hallier f	Apocynaceae	Mototoko	de Wilde J.J.F.E. 8371	Bark	Barks are used to increase dog’s hunting performance	0.11
*Hypselodelphys zenkeriana (K.Schum.) Milne-Redh*	Marantaceae	Ligombe	Breteler F.J. 1043	Root	Roots are used to bring luck	0.11
*Leplaea cedrata *(A.Chev.) E.J.M.Koenen & J.J.de Wilde	Meliaceae	Mbegna	Mpom B. 24	Bark	Barks are used to get courage	0.11
*Lonchitis hirsuta *L	Lonchitidaceae	Gobouma		Bark	Barks are used to attract animals	0.11
*Maesopsis eminii Engl*	Rhamnaceae	Kanga		Bark	Barks are used to bring luck	0.11
*Mangifera indica *L	Anacardiaceae	Manguier	Mpom B. 22	Root	Roots are used to increase dog's hunting performance	0.11
*Maranthes glabra (Oliv.) Prance*	Chrysobalanaceae	Mbokandja	de Wilde W.J.J.O. 2652	Stem	Stems are used as trap lever and to make spear	0.11
*Meiocarpidium oliverianum (Baill.) D.M.Johnson & N.A.Murray*	Annonaceae	Mabelengue	Letouzey R. 10,153	Stem	Stems are used to make spear	0.11
*Milicia excelsa (Welw.) C.C.Berg*	Moraceae	Bangui	Thomas D.W. 6869	Bark	Barks are used to bring luck	0.11
*Morelia senegalensis A.Rich. ex DC*	Rubiaceae	Edjé	Letouzey R. 2886	Stem	Stems are used as trap lever	0.11
*Musa sp.*	Musaceae	Moundédé	Raynal J. 10,776	Fruit	Fruits are used to attract animals	0.11
*Nicotiana tabacum L*	Solanaceae	Ndako	Swarbrick 213	Leaves	Leaves are used to attract animals	0.11
*Omphalocarpum sp.*	Sapotaceae	Ngwadjala	Thomas D.W. 7917	Leaves	Leaves are used to get courage	0.11
*Pauridiantha pyramidata (K.Krause) Bremek*	Rubiaceae	Ngwa'a	Letouzey R. 1696	Leaves	Leaves are used as fish poison	0.11
*Persea americana Mill*	Lauraceae	Avocatier	Ekema 09	Leaves	Leaves are used to have luck	0.11
*Piper umbellatum L*	Piperaceae	Mbebelembe	Nkongmeneck B.-A. 877	Stem	Stems are used to make fire	0.11
*Raphia sp.*	Arecaceae		Letouzey R. 15,260	Stem	Stems are used to make baskets to carry catches and to make lobster pot	0.11
*Santiria trimera (Oliv.) Aubrév*	Burseraceae	Ebaba, Libaba	Letouzey R. 4684	Bark	Barks are used to bring luck and to increase dog's hunting performance	0.11
*Scottellia klaineana Pierre*	Achariaceae	Kpwomboseko	Letouzey R. 5057	Bark	Barks are used to have luck	0.11
*Sida rhombifolia L*	Malvaceae	Ntadanda	Breteler F.J. 278	Leaves	Leaves are used to bring luck	0.11
*Sterculia oblonga *Mast	Malvaceae	Mboyo	Thomas D.W. 4945	Bark	Barks are used to bring luck	0.11
*Trichosypha sp.*	Anacardiaceae	Ngoyo'o	Nemba 15	Bark	Barks are used to bring luck	0.11
*Not identified*		Adama		Bark	Barks are used as fish poison	0.68
*Not identified*		Molombi		Root, bark, stem, leaves	Roots, barks, stems and leaves are used to become invisible	0.68
*Not identified*	*Marantaceae*	Gwasa'a	Lowe J. 3107	Leave	Leaves are used for chasing water during dam fishing and for packaging products	0.34
*Not identified*		Mentem		Stem	Stems are used as trap lever	0.34
*Not identified*		Gbwo		Fruit	Fruits are used to increase dog's performance	0.23
*Not identified*		Meningoumbe		Stem	Stems are used to make lobster pot	0.23
*Not identified*		Alokou		Bark, leave	Barks and leaves are used as fish poison	0.23
*Not identified*		Moloundou		Bark	Barks are used to increase dog' hunting performance and to get courage	0.23
*Not identified*		Ngalé		Bark	Barks are used as fish poison	0.23
*Not identified*		Mindoundoun		Stem	Stems are used as trap lever	0.23
*Not identified*		Djouendjel		Stem	Stems are used as trap lever	0.11
*Not identified*		Etoa		Stem	Stems are used as fish poison	0.11
*Not identified*		Ethnospecies 1			Used to get courage	0.11
*Not identified*		Mapembey		Leaves	Leaves are used to bring luck	0.11
*Not identified*		Npossa		Leave	Leaves are used as container	0.11
*Not identified*		Ethnospecies 2			Used to make baskets to carry catches	0.11
*Not identified*		Bokobogwamé		Bark	Barks are used to increase dog’s hunting performance	0.11
*Not identified*		Ethnospecies 3		Stem	Stems are used as trap lever	0.11
*Not identified*		Kouma			Used to become invisible	0.11
*Not identified*		Gkouelo		Stem	Stems are used to make spear	0.11
*Not identified*		Gwandjaka		Leaves	Leaves are used to bring luck	0.11
*Not identified*		Gwi		Bark	Barks are used to increase dog’s hunting performance	0.11
*Not identified*		Kouogouo		Stem	Stems are used to make lobster pot	0.11
*Not identified*		Kpobala		Leaves	Leaves are used to increase dog's hunting performance	0.11
*Not identified*		Ethnospecies 4			Used to increase dog's hunting performance	0.11
*Not identified*		Adia		Leaves	Leaves are used to increase dog's hunting performance	0.11
*Not identified*		Lo'o		Stem	Stems are used as trap lever	0.11
*Not identified*		Ma'a		Stem	Stems are used to bring luck	0.11
*Not identified*		Messini		Bark	Barks are used to bring luck	0.11
*Not identified*		Metonga		Bark	Barks are used to become invisible	0.11
*Not identified*		Moboumso		Stem	Stems are used to bring luck	0.11
*Not identified*		Mokope		Bark	Barks are used to get courage	0.11
*Not identified*		Mongola		Stem	Stems are used to take animals out of the hole	0.11
*Not identified*		Mototombo		Stem	Stems are used as trap lever	0.11
*Not identified*		Nkenkeguili		Leaves	Leaves are used to bring luck	0.11
*Not identified*		Bekesso		Stem	Stems are used as trap lever	0.11
*Not identified*		Npoh		Bark	Barks are used to bring luck	0.11
*Not identified*		Nto'o		Bark	Barks are used to become invisible	0.11
*Not identified*		Simbo		Stem	Stems are used to become invisible	0.11
*Not identified*		Ethnospecies 5		Stem	Stems are used as trap lever	0.11
*Not identified*		Ethnospecies 6		Stem	Stems are used to bring luck	0.11
*Not identified*		Ethnospecies 7			Used as fish poison	0.11
*Not identified*		Ethnospecies 8			Used to bring luck	0.11
*Not identified*		Ethnospecies 9			Used to get courage	0.11
*Not identified*		Ethnospecies 10			Used to bring luck	0.11
*Not identified*		Ethnospecies 11			Used to get courage	0.11
*Not identified*		Ethnospecies 12			Used to make lobster pot	0.11
*Not identified*		Ethnospecies 13			Used to bring luck	0.11
*Not identified*		Bolombi			Used to become invisible	0.11
*Not identified*		Eke		Stem	Stems are used to make baskets to carry catches	0.11
*Not identified*		Gwabotouga		Leaves	Leaves are used to bring luck	0.11
*Not identified*		Ngokele		Bark	Barks are used to increase dog’s hunting performance	0.11
*Not identified*		Eloukou		Bark	Barks are used to increase dog’s hunting performance	0.11
*Not identified*		Gomabolo		Bark	Barks are used to increase dog’s hunting performance	0.11
*Not identified*		Ethnospecies 14			Used to make baskets to carry catches	0.11
*Not identified*		Mbaté		Bark	Barks are used to attract animals	0.11
*Not identified*		Molondo		Bark	Barks are used to increase dog’s hunting performance	0.11
*Not identified*		Njombo		Bark	Barks are used to get courage	0.11
*Not identified*		Ethnospecies 15			Used to increase dog's hunting performance	0.11
*Not identified*		Ethnospecies 16			Used to get courage	0.11

**Fig. 4:**
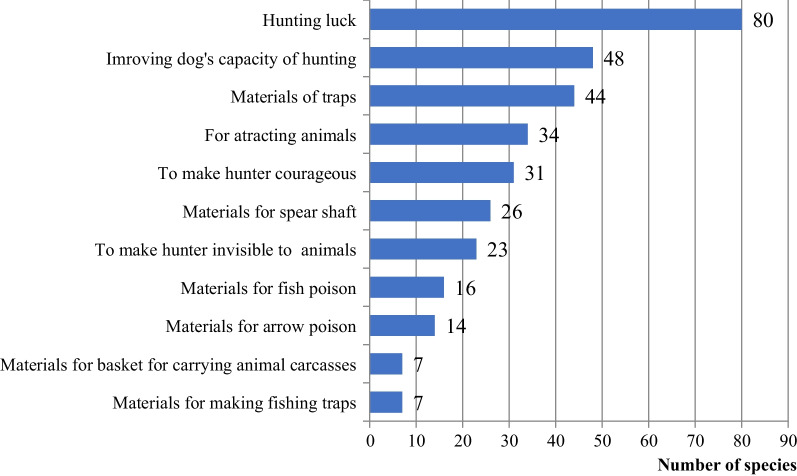
Numbers of plants and their usages for hunting and fishing

These plants were used for a variety of purposes including materials for making traps, baskets for transportation, arrow poison, fish poison, etc. (Fig. 4). Others were used in ritual practices aimed at becoming invisible to dangerous animals, attracting animals, or to have luck when going out for a hunting expedition. Others were used directly by the hunter to be courageous or to chase away dangerous animals. The use of dogs was another technique of hunting widely used in the region, as well as in other continents. A variety of plant species were used for improving the scent and other abilities of dogs.

### Variation in the ethnobotanical knowledge for hunting with age and gender

The comparative analysis of the extent of plant knowledge among respondent groups showed no significant difference between men and women, which indicates there is no gender-based pattern in the knowledge of plants used in hunting practices. The 75 women interviewed cited 122 plants, with a ratio of 1.62 plants per respondent, while the 90 men cited 174 plants, with a ratio of 1.92 per respondent. Concerning the effect of age, the largest number of plants was cited by the respondent group of 20–30 years old (109 plant species cited by 55 respondents). This might be partly explained by the larger size of this group interviewed. The highest ratio of plant citation per respondent is recorded in the age group 50–60 years where a total of 14 respondents cited as many as 73 species, with a ratio of 5.21 per respondent (Table 3).

**Table 3:** Score of citation of plants by age groups

Age group	No. of respondents	No. of plants cited	Ratio (number of plant citation/respondent)
10–20 years	36	89	2.47
20–30 years	55	109	1.98
30–40 years	27	75	2.78
40–50 years	19	59	3.11
50–60 years	14	73	5.21
+ 60 years	15	47	3.13

The value of the NNESS similarity index between lists of plants cited by men and women was 0.68, indicating a certain degree of commonality between the two groups.

The plants listed by aged members (+ 60 years) were significantly different from those of other age groups, as this is shown by the NNESS values of 0.39, which means the most-aged groups cited fewer common species (Table 4).

**Table 4:** NNESS similarity indices between the lists of plants cited by age groups

NNESS(k = 50)						
Age	10–20 years	20–30 years	30–40 years	40–50 years	50–60 years	+ 60 years
10–20 years	1.00					
20–30 years	0.69	1.00				
30–40 years	0.64	0.69	1.00			
40–50 years	0.57	0.65	0.64	1.00		
50–60 years	0.53	0.56	0.60	0.59	1.00	
Above 60	**0.39**	**0.47**	0.51	0.54	**0.40**	1.00

Values in bold show pairs of age groups having greater differences in their lists of plants cited

The group of 10–20 years old and that of above 60 years shared only 25 common species. Of the 89 species cited by younger groups, 53 were not cited by the group of above 60 years. Among those non-common species, the most frequent are: *Diospyros crassiflora* Hiern*, Massularia acuminata (*G.Don) Bullock ex Hoyle*, Acacia pennata* (L.) Willd.*, Brenania brieyi* (De Wild.) E.M.A.Petit*, Cleistopholis glauca* Pierre ex Engl. & Diels*, Diospyros hoyleana* F.White*, Heisteria zimmereri* Engl.*, Strophanthus gratus* (Wall. & Hook.) Baill.*, Xylopia sp., Albizia sp., Alchornea floribunda* Müll.Arg.*, Cleistopholis patens* (Benth.) Engl. & Diels*, Manniophyton fulvum* Müll.Arg.*, Tabernaemontana crassa* Benth. and *Terminalia superba* Engl. & Diels*.* On the other hand, of the 47 species cited by respondents above 60 years, 26 were not known by younger respondents aged between 10 and 20 years. They consist of the species like *Landolphia heudelotii* A.DC.*, Erythrophleum suaveolens* (Guill. & Perr.) Brenan*, Tetrapleura tetraptera* (Schumach. & Thonn.) Taub*., Barteria nigritiana* Hook.f. and *Manihot esculenta* Crantz*.*

## Discussion

In the study villages, the importance of plants in hunting and fishing practices is well established. The plant species recorded as used in hunting practices are taxonomically quite diverse. Some have been cited more frequently than others. A previous botanical survey in this peripheral site of the Dja biosphere reserve has reported wider distribution and abundance of these species in the study area [35]. Given this availability, the Baka peoples in the study villages may therefore preferentially use the plants that are readily available in their neighbourhood for hunting purposes. Hunting and fishing practices recorded in the studied villages are in line with those previously reported by other researchers [12, 36–39]. Some of the species recorded as used in fishing and hunting practices have been described to have similar uses in the previous ethnobotanical literature in central Africa [2, 8]. The use of Marantaceae (*Megaphrynium macrostachyum and Ataenidia conferta (Benth.)* Milne-Redh.) in dam fishing was previously reported by [39, 40] and [4]. Some of the recorded plants like *Drypetes* spp. and *Greenwayodendron suaveolens* have also been reported as used for making spears or snares by Baka hunter-gatherers in Cameroon [15]. The use of the barks of *Zanthoxylum gilletiim* (De Wild.) P.G.Waterman, *Turraeanthus africanus* (Welw. ex C. DC.) Pellegr. and the fruits of *Microcos coriacea* (Welw. ex C. DC.) Pellegr. as fish poison, the application of *Desbordesia glaucescens* (Engl.) Tiegh. to improve the chance of catching more animals as well as the use of *Tetrapleura tetraptera* (Schumach. & Thonn.) Taub. and *Aframomum melegueta* K. Schum. to improve the performance of dogs in hunting have been documented by several authors in Central Africa as well as in West Africa and south America [39–41]. Natural poisons derived directly from plants have been used in fishing for millennia [42, 43], poisonous ingredients are pounded and thrown into a pool or dammed sections of a small river. After a time, which varies according to conditions the fish begin to rise to the surface of the water and can readily be taken by hand [17]. Several studies have shown that these poisons have no effect on human health, humans can digest it relatively safely [17, 44]. Although the consumption of preys killed with the natural poison has no effect on human health, [45] recorded an anaesthetic effect on limbs and roughness of skin of people who wade into streams to collect fish poisoned with *Tephrosia vogelii* Hook.f. These considerations should definitely be taken into account in the spread of these practices. The major problem in using fishing poisons is the massive destruction of aquatic organisms. Natural fish poisons paralyse or kill fish; sometimes, they kill other aquatic organisms; therefore, this practice has been banned in many contexts due to the ecological damage it can cause [17, 46].

Results of this study show that there is an uneven distribution of ethnobotanical knowledge for hunting within studied communities. Although the ratio of plant citation per respondent was higher among aged groups, similarity analysis shows that younger respondents cited a larger number of plant species used for hunting and fishing and that they named many plants that were not cited by the elders. These observations are contrary to those of [47] who reported that only adults can master hunting or medicinal plant knowledge. For instance, ethnobotanical knowledge acquisition process among the studied community involves plural channels and sources. It is acquired through contact with the people with different background, such as family members, schoolmates and neighbours of other ethnic groups, associated with their socialization process. Younger members of the community engage in a variety of game and subsistence-related activities. The Baka living on the periphery of the Dja biosphere are sedentary along the roads, so the young Baka interact with many other youths including Bantus of the similar age, and through this process of social contacts, there is a dynamic of recompositing of their original traditional knowledge acquired from their family members. In these dynamics, they accumulate their own ethnobotanical knowledge that extends beyond the scope of knowledge acquired within a narrow range of their own family. This observation is consistent with previous findings by [20] on the Baka in the same region of East Cameroon. Moreover, it is clearly established that traditional ecological knowledge transmission most often occurs between older and younger generations, or vertical instruction; it can also occur through more horizontal interactions between peers and through oblique transmission from non-familial mentors [48]. These different knowledge acquisition pathways were qualified as “multiple-stage learning process” by [49, 50].

From the similarity indices among the plant lists of different groups of respondents, it is shown that 68% of plant species recorded are shared between men and women. This commonality, however, masks finer gender differences. The social organization of the hunting activities involves both men and women who share the responsibilities. Although some of the hunting activities are performed by men, many of the rituals during which plants are used are performed by women and particularly virgin girls. In fact, from the discussion with respondents, it was revealed that according to the traditional beliefs within Baka society, the prayers and words sent to the ancestors to ask for luck require that the person sending the prayers be “pure”. For instance, virgin girls in Baka society are a symbol of good moral behaviour and conduct and thus of purity, as they have not yet engaged in sexual relationships considered as a sin that defiles the body of the person. Hence, they believe that their ancestors would be happy, answering to the will of virgin girls than that of any other person.

There are cases where women, especially elder members, play a key role in the transmission of ethnobotanical knowledge for hunting to men.

In such a context, ethnobotanical hunting knowledge acquisition within the studied communities might involve several individual and collective factors associated with their socialization process.

## Conclusion

This study was conducted to document the traditional knowledge of plants used in hunting practices among the Baka community members in Eastern Cameroon. Results showed a taxonomic list of 176 species used by the studied populations. In the practice of hunting or fishing, these plants are used for a variety of purposes including as materials for making traps or baskets for transportation, arrow or fish poison, traditional medicine used by the hunter to be courageous or by the dogs to improve their scent and ability for hunting, materials for ritual practices performed to become invisible to dangerous animals, attract animals, to chase away dangerous animals or to have luck when going out for a hunting expedition.

Although female Baka are traditionally more active in fishing, they are knowledgeable of plants used for hunting as well. The ethnobotanical knowledge of using plants for hunting did not vary significantly with gender, but showed some variation among age groups, with younger members citing more plants than the elder. For further investigations, a comparative study with neighbouring Bantu communities will be important to understand the dynamics of inter-community knowledge exchange.

## Data Availability

The data sets used and/or analysed during the current study are available from the corresponding author upon reasonable request.
